# Exploring factors influencing depression among Polish nurses during the COVID-19 pandemic

**DOI:** 10.3389/fpubh.2023.1272082

**Published:** 2023-09-14

**Authors:** Kamila Rachubińska, Mariusz Panczyk, Marcin Sygut, Przemysław Ustianowski, Elżbieta Grochans, Anna Maria Cybulska

**Affiliations:** ^1^Department of Nursing, Faculty of Health Sciences, Pomeranian Medical University in Szczecin, Szczecin, Poland; ^2^Department of Education and Research in Health Sciences, Faculty of Health Science, Medical University of Warsaw, Warsaw, Poland; ^3^Department of Social Medicine and Public Health, Chair of Social Medicine, Pomeranian Medical University in Szczecin, Szczecin, Poland

**Keywords:** anxiety, COVID-19, depression, mental health, insomnia, nursing, pandemic

## Abstract

**Objectives:**

The COVID-19 pandemic has been recognized as an international public health emergency. The aim of our study was to identify contributors to nurses’ depression.

**Methods:**

This survey-based study was conducted in the Pomeranian Medical University Hospital no. 1 in Szczecin and involved 207 nurses. The following standardized research instruments were applied: the World Assumptions Scale, the Athens Insomnia Scale, the Impact of Event Scale - Revised, the Patient Health Questionnaire-9, the Generalized Anxiety Disorder, the Perceived Stress Scale, and a questionnaire of our own authorship.

**Results:**

The study showed that 72.95% of the subjects experienced severe stress, and 40.58% suffered from insomnia. In addition, 65.7% of the respondents had anxiety symptoms of varying degrees of severity, and 62.8% had depressive symptoms of mild to severe severity. The mean score on the IES-R scale, reflecting a psychological impact of the COVID-19 pandemic, was 34.25. The COVID-19 pandemic affected the psychological health of medical staff, particularly through increased stress and anxiety symptoms. Anxiety levels and insomnia significantly affect the prevalence of depression among nurses.

**Conclusion:**

The COVID-19 pandemic has been recognized as an international public health emergency. The COVID-19 pandemic affected the psychological health of medical staff, particularly through increased stress and anxiety symptoms. It is important to conduct further research after the COVID-19 pandemic has ended.

## 1. Introduction

WHO expressed concern about the COVID-19 consequences for mental health and psychosocial well-being around the globe (1). Early in the pandemic, it was observed that isolation or quarantine significantly affected the usual activities or livelihood of many people, which could result in increased levels of anxiety or depression, increased insomnia, alcohol or drug abuse, self-harm, or even suicidal behavior (2–5). Due to the increasing number of COVID-19 cases, a high number of deaths, inefficient health systems around the world, people felt the fear of infection, death, complications and lack of contact with other people often belonging to the immediate family who may have been infected. In addition to health problems, the COVID-19 pandemic also brought significant changes in many areas of social life: the closure of schools and workplaces, economic problems, as well as isolation from loved ones and reduced social contact. There was the stress of being in quarantine and prolonged isolation. Stress was also a factor in insomnia and depressive behavior. From the very beginning, health care workers were a group particularly vulnerable not only to infection, but also a strong impact of many stressors associated with the attempts to control the pandemic. Factors negatively affecting the psychological functioning of this occupational group included excessive workload, risk of infection, exhaustion, overwork, isolation, lack of adequate personal protective equipment, sleeplessness, and deprivation of contact with loved ones. Moreover, asymptomatic patients, who could unknowingly spread the virus, posed a particular risk of exposure to health care workers (6). All these may result in worse work performance (7). Fighting an emergency situation, often putting in excessive hours, working with a high risk of infection from patients can lead to mental health problems such as stress, anxiety and depression.

The literature review indicates that are a lot of causes of depressive symptoms among healthcare professionals. Nurses regularly experience a variety of factors contributing to depressive symptoms (e.g., work-related stress, anxiety). The assessment of the impact of stress on the work and health of nurses is an important element in preventing many threats, including burnout. Poor mental health among nurses may hinder their professional performance, and decrease the energy or work efficiency of nurses. Moreover, it has a considerable effect on the quality of patient care, which ultimately has a negative impact on patient outcomes, increased incidences of practice errors, and loss of compassion for patients (8–10).

Emerging chronic stressors, can destabilize an individual’s functioning. As proven, chronic stress plays an important role in the pathogenesis of depression. In addition, it can have a destabilizing effect on the immune system, causing changes typical of inflammation. Similar changes are found in depressed patients. The COVID-19 pandemic is a strong psychosocial stressor, and the restrictions imposed by many governments further increase social stress. It seems that the main stressors in the era of the pandemic are fear of getting sick (both one’s own and one’s loved ones’), fear of destabilizing one’s economic situation, and anxiety related to socioeconomic uncertainty. Mood disorders, anxiety and sleep problems - common symptoms of chronic stress - can be part of adaptive disorders, but are also an immanent part of depressive disorders. Exposure to deep, prolonged stress during the COVID-19 pandemic will have a negative impact on the mental health of the public, including health care workers. Nurses who have direct contact with COVID-19 patients are particularly vulnerable to symptoms such as depression, anxiety, stress and poor sleep quality, and their mental health may require special attention. In the fight against the COVID-19 pandemic, medical workers often have to live with the knowledge of the risk of infection. They are often under-equipped with protective gear, overworked, and exhausted. Also sleep-deprived, isolated and deprivation of contact with loved ones intensifies adverse symptoms. The difficult situation often negatively affects their mental stress levels, which can result in a decrease in the quality of their work. In the face of these risks, it is necessary to implement a comprehensive mental health plan, especially among workers who have direct contact with the infected (7, 9, 10).

Nurses providing care to patients with COVID-19 have been found to often complain about significant work-related psychological burden and somatic symptoms, such as increased irritability, muscle tension, nervous breakdown, difficulty sleeping, nightmares, gastrointestinal problems, palpitations, shortness of breath, chest pain, and dizziness (11). Moreover, the medical personnel were much more likely than the hospital administrative staff to fear the lack of control of virus spread, contagion, lack of personal protective measures, felt more lonely as a result of isolation from loved ones, and were more frustrated with the results of treatment (12). It is worth noting that social support was one of the factors that reduced anxiety or stress levels and had a positive impact on well-being and medical staff effectiveness (13). Other variables that reduced stress levels among health care workers included: no infections among staff after strict protective measures, increasing knowledge, protective equipment provided by the hospital, improved patient condition, no illnesses among family members, decreasing number of new infections, no overtime, sense of humor, free meals, and compensation for infection risk (14).

The literature review showed that those working in direct proximity of COVID-19 patients, working in high-risk units, nurses and women are at significant risk of mental health issues and should be a priority in staff support within hospitals (9). Moreover, studies have shown that working in the pandemic have led to feelings of threat, uncertainty, and fatigue among hospital healthcare professional. Many reviews have documented a range of mental health issues among hospital healthcare professionals during the pandemic, such as depression (15, 16), anxiety (17), post-traumatic stress disorder (16) and insomnia (18, 19). Due to the negative influence of COVID-19 pandemic on the mental health of nurses, identification of the causes and prevalence of depressive symptoms, stress, and anxiety in the workplace among nurses taking care of patients diagnosed with COVID-19 is necessary (8–10).

The aim of our research was to understand the effects of the COVID-19 pandemic on depressive symptoms among Polish nurses. Moreover, we wanted to investigate the risk and predictive factors of depressive symptoms among Polish nurses during the pandemic COVID-19. The investigation presented here was performed as part of a larger project and was intended to help assess the need for psychological support and to enable the selection of psychological variables influenced by the pandemic.

In order to address these gaps in the literature, our study will analyze the factors influencing depression among nurses during the COVID-19 pandemic.

We hypothesized that:

*H1*: Nurses were repeatedly exposed to traumatic stress at their workplace or there were at high risk of experiencing anxiety, insomnia, and depressive symptoms during the COVID-19 pandemic.

*H2*: There are many risk factors linked to depression during the pandemic COVID-19 for example: stress, anxiety, or insomnia. Protective factors for depression are good problem-solving skills, a high level of self-esteem, and self-behavioral control.

We formulated the following research questions:

- How does the pandemic COVID-19 influence the level of stress among nurses?
- What are nurses’ most common risk factors of depression that significantly affect their mental health?

## 2. Materials and methods

### 2.1. Research design

The study was carried out in the Pomeranian Medical University Hospital no. 1 in Szczecin. The research was conducted among 207 nurses having direct contact with people infected with SARS-CoV 2. The selection of the group was random. No randomizing tool was used. It was based on self-reported respondents who met the inclusion criteria as described in the limitations. The research was based on a survey performed using a questionnaire technique. Due to epidemic reasons, the study was conducted using online tools in Polish.^1^ The research was in line with the Declaration of Helsinki requirements. The respondents were informed that the survey was anonymous and that they could opt-out at any stage.

All procedures performed in studies involving human participants were in accordance with the ethical standards of the institutional and/or national research committee and with the 1964 Helsinki Declaration and its later amendments or comparable ethical standards. The study was approved by the Bioethics Committee of the Pomeranian Medical University in Szczecin (KB-0012/102/12/2013).

Participants were qualified for the study based on the same inclusion criteria as in the previous research: current nursing license, age above 18 years, informed consent to participate in the study, and correctly completed questionnaires. The exclusion criteria were: no consent to participate in the study, incorrectly completed questionnaires, another health professional (e.g., phisician, paramedic).

### 2.2. Surveys

The following standardized research tools were used to identify factors contributing to nurses’ depression during the COVID-19 pandemic:

- The Athens Insomnia Scale (AIS)- is a scale consisting of 8 test items assessing: falling asleep, waking up at night, waking up too early in the morning, sleep duration, quality of sleep, mood the next day, psychophysical performance the next day, and daytime sleepiness. The Athens Insomnia Scale is the first tool to assess insomnia-related symptoms that has Polish validation. Each question is assigned four possible responses scored from 0 to 3. The total score fall between 0 and 24, where higher scores indicate poorer sleep quality (Cronbach’s alpha = 0.90) (20, 21).
- The Generalized Anxiety Disorder-7 (GAD-7)-is a seven-point, validated scale, designed to assess the severity of anxiety symptoms and to screen for generalized anxiety disorder. It is a self-assessment scale in which the patient marks the frequency of some anxiety symptoms during the last 2 weeks (from 0 to 3 points). GAD-7 can also be used for screening for panic disorder, post-traumatic stress disorder and social anxiety disorder. The advantages of the questionnaire are its simplicity and short duration of the survey. A version of the GAD7 was translated into Polish by the MAPI Research Institute.^2^ The obtained Cronbach’s alpha reliability coefficient for the questionnaire in our study was *α* = 0,94 (22).
- The Impact of Event Scale - Revised (IES-R)-is used to measure subjective post-traumatic symptoms. The Impact of Event Scale is a Polish adaptation of the revised version of the Impact of Event Scale (IES-R) by Weiss and Marmar, adapted to Polish conditions by Juczyński and Oginska-Bulik. The scale captures the three dimensions of PTSD: intrusion, arousal, and avoidance. This tool includes 22 statements concerning symptoms of stress occurring during 7 days following a traumatic event. It describes 3 dimensions of stress: intrusion, which involves the recurrence of trauma-related images, dreams, and thoughts; arousal, which invoves higher body activity; and avoidance - thoughts, places, and conversations associated with the trauma (Cronbach’s alpha = 0.92) (23, 24).
- The Patient Health Questionnaire-9 (PHQ-9)-is used in the diagnosis of mental disorders based on the Diagnostic and Statistical Manual of Mental Disorders (DSM-IV) criteria. The PHQ-9 consists of nine questions, or rather the DSM-IV depression criteria. It enables both the diagnosis of depression and the assessment of the severity of its individual symptoms. The Polish version is available at the MAPI Research Institute: www.phqscreeners.com (Cronbach’s alpha = 0.77) (25).
- The Perceived Stress Scale (PSS-10)-was developed by Cohen, Kamarck and Mermelstein and its Polish version by Juczyński and Ogińska-Bulik. It serves for measuring the severity of stress associated with the respondent’s life situation over the past month. The scale includes 10 questions concerning subjective feelings related to personal problems, events, and behaviors as well as ways of dealing with them. The respondent answers each question by assigning a value from 0 to 4, where 0 means: never and 4: very often. The scale is used to examine adults, healthy and sick (Cronbach’s alpha = 0.86) (26, 27).
- The World Assumptions Scale (WAS) is a self-report questionnaire consisting of 32 items regarding assumptions about the world. WAS is a questionnaire to assess beliefs and their changes due to the occurrence of a critical life event in a person’s life, Polish adaptation by Załuski and Gajdosz. The scale distinguishes the following 3 major groups: kindness of the surrounding world: (benevolence of the surrounding world, benevolence of people); relevance and explain ability of life events (control of negative events, fairness, randomness) and human valence (self-value, happiness, control of own behavior). Answers are given on a 6-point scale ranging from 1 (strongly disagree) to 6 (strongly agree). (Cronbach’s alpha = 0.816) (28, 29).
- The self-survey questionnaire included questions about sociodemographic data, well-being during the last 14 days, disturbing symptoms and contact with someone with confirmed COVID-19.

### 2.3. Statistical analysis

The descriptive statistics was used to present the Quantitative and categorical (ordinal and nominal) variables. Quantitative variables were described using the following measures: mean and median (measures of central tendency), standard deviation, quarterly interval, and coefficient of variance (measures of dispersion). Frequency (N) and percentage (%) were used to describe the categorical variables.

A multivariate linear regression model was used to estimate the influence of selected factors on the prevalence of depressive symptoms (according to PHQ-9) among nurses. The parameters of the regression model were estimated by the Least Squares Method. The following rates were determined for each predictor in the regression model: unstandardized (b) and standardized regression (βstand.) coefficient with 95% confidence interval (CI). The adjusted R-squared (R2adj.) was calculated to determine the proportion of the variance for a dependent variable that’s explained by independent variables.

All calculations were done with STATISTICA 13.3 (TIBCO Software, Palo Alto, California, United States). Statistical significance was set as *p* < 0.05.

## 3. Results

### 3.1. Brief characteristics of the healthcare professionals

A total of 312 respondents working directly with patients diagnosed with COVID-19 were invited to participate in the survey. Only 207 nurses correctly completed the surveys (completion rate: 66%), whose mean age was 37.8 years (SD = 11.98). Most of the respondents were female (83.09%), with higher education (79.23%), in a formal relationship (52.66%), and more than half (57%) of the respondents came from a city of more than 100,000 inhabitants and not working in shifts (68.6%). In addition, 19.32% were quarantined due to COVID-19 and 42.51% of the respondents had access to psychological support. The vast majority of respondents had direct contact with someone with confirmed COVID-19 infection within 4 weeks of completing the survey questionnaire, and 35.7% had indirect contact (involving personal protective equipment) with a person with confirmed COVID-19 infection (Table 1).

**Table 1:** Sociodemographic variables.

Variables		*N*	%
Gender	Female	256	79.3
	Male	67	20.7
Marital status	Formal relationship	153	47.4
	Informal relationship	78	24.1
	Single/divorced/widowed	92	28.5
Education	Secondary	57	17.6
	Post-secondary	14	4.3
	Higher (I)	99	30.7
	Higher (II)	141	43.7
	Higher (III)	12	3.7
Place of residence	Village	67	20.7
	City with up to 10,000 people	16	5.0
	City with 10,000–100,000 people	56	17.3
	City with over 100,000 people	184	57.0
Children	No	160	49.5
	Yes	155	48.0
	Pregnancy	8	2.5

N Frequency, % percentage.

### 3.2. Analysis of perceived stress, assumptions about the world and prevalence of anxiety, depression, insomnia among nurses during the SARS-CoV-2 coronavirus pandemic

The study analyzed selected mental health variables (sleep problems, anxiety, stress, depression).

The mean score on the Athens Insomnia Scale was 6.71 (SD-4.39); 59.42% of the respondents did not have insomnia. In the case of anxiety assessed by the Generalized Anxiety Questionnaire (GAD-7), the mean score was 7.12 (SD- 5.41). 65.7% of the respondents obtained total scores indicating the presence of anxiety symptoms of varying severity. In addition, the vast majority (72.9%) of the subjects had high levels of stress according to the PSS-10, with the mean sten scores of 7.14 (SD = 1.56). For depressiveness according to the PHQ-9 scale, the mean score was 7.77 (SD-6.20), and 37.2% of the respondents did not have symptoms of depression. The psychological impact of the COVID-19 pandemic, measured by the IES-R, was reflected by a mean score of 34.25 (SD = 19.65). Analysis of the three dimensions of post-traumatic stress disorder measured by this scale revealed that the mean scores for intrusion, arousal and avoidance were 12.34, 11.58 and 12.12, respectively. The study also analyzed the results obtained in The World Assumptions Scale, which showed that the highest mean score in the study group was for happiness (*M* = 17.52), control of one’s own behavior (*M* = 16.77) and kindness to people (*M* = 15.88), while the most dominant group assessed in the WAS scale was human worthiness (*M* = 50.48; Tables 2, 3).

**Table 2:** Descriptive statistics of: insomnia according to AIS, generalized anxiety according to GAD-7, Impact of Events Scale (IES-R), depressiveness according to PHQ-9, stress according to PSS-10 and The World Assumptions Scale in the whole group of subjects.

Variables	*M*	SD	IQR/2	Min-Max	CV [%]
AIS (sum of pts)	6.71	4.39	3.50	0–22	65.41
GAD-7 (sum of pts)	7.12	5.41	4.00	0–21	76.03
IES-R (sum of pts)	34.25	19.65	15.50	0–88	57.38
*IES-R, Intrusion*	12.34	7.78	6.00	0–32	63.05
*IES-R, Stimulation*	11.58	6.26	4.50	0–28	54.05
*IES-R, avoiding*	12.12	6.31	4.50	0–28	52.10
PHQ-9 (sum of pts)	7.77	6.20	4.00	0–27	79.78
PSS-10 (sten)	7.14	1.56	1.00	1–10	21.80
WAS (sum of pts)	120.71	13.25	9.50	77–157	10.97
*WAS, The kindness of people*	15.88	3.19	2.00	7–24	20.11
*WAS, The kindness of the world*	12.50	3.23	2.00	4–21	25.86
*WAS Negative Event Control*	15.82	3.55	2.50	4–24	22.47
*WAS Justice*	15.75	3.91	2.50	4–24	24.83
*WAS, Randomness*	15.00	3.71	2.50	4–22	24.71
*WAS, Own value*	12.33	3.87	3.00	4–24	31.35
*WAS Happiness*	17.52	3.84	2.50	8–24	21.94
*WAS, Controlling one’s own behavior*	16.77	3.20	2.00	4–24	19.06
*WAS, The kindness of the surrounding world*	34.23	6.58	4.50	14–51	19.22
*WAS, The meaningfulness and explainability of life events*	33.42	5.75	3.50	11–50	17.22
*WAS Human Values*	50.48	7.23	5.50	27–67	14.32

AIS, Athens Insomnia Scale; GAD-7, generalized anxiety disorder; IES-R, Impact of Event Scale - Revised; PSS-10, The Perceived Stress Scale; WAS, World Assumptions Scale M- mean Me- median SD- standard deviation IQR/2- quarterly interval; CV- Coefficient of Variation.

**Table 3:** Prevalence of: insomnia by AIS, generalized anxiety by GAD-7, depression by PHQ-9, and stress by PSS-10 in the study group of nurses.

Variables		*N*	%
AIS	No insomnia	123	59.42
	Insomnia	84	40.58
GAD-7	No anxiety	71	34.30
	Mild anxiety	76	36.71
	Moderate anxiety	41	19.81
	Severe anxiety	19	9.18
PSS-10	Low (1–4 steny)	13	6.28
	Moderate (5–6 stena)	43	20.77
	High (7–10 stena)	151	72.95
PHQ-9	Absence of depression	77	37 0.20
	Mild depression	64	30 0.92
	Depression	66	31 0.88

N Frequency, %, percentage, AIS, Athens Insomnia Scale, GAD-7, generalized anxiety disorder; PSS-10, The Perceived Stress Scale; PHQ-9- Patient Health Questionnaire-9.

### 3.3. Analysis of the relationship between mental health variables (anxiety, stress, insomnia) and the level of depression among nurses during the SARS-CoV-2 coronavirus pandemic

This study analyzed the effect of selected mental health variables on the level of depression among nurses during the SARS-CoV-2 coronavirus pandemic.

Based on the results, model 1 explained more than 66% of the variability in the depression variable according to PHQ-9 [*F*(5, 201) = 81.446, *p* < 0.001]. Respondents with higher severity of generalized anxiety according to the GAD-7 scale were characterized by higher levels of depressiveness (*p* < 0.001). Moreover, subjects with insomnia symptoms confirmed by the AIS had more severe depression according to the PHQ-9 than their counterparts without such symptoms (*p* < 0.001; Table 4).

**Table 4:** Influence of insomnia by AIS, anxiety by GAD-7, stress by PSS-10, World Assumptions Scale on the prevalence of depressive symptoms among nurses according to PHQ-9 (Model 1,2,3).

Factors	*b*	−95% CI	+95% CI	*t*	*p*
MODEL 1					
Intercept	1.266			0.501	0.617
AIS (insomnia vs. no insomnia)	1.571	0.155	0.344	5.204	0.000
GAD-7 (total)	0.613	0.426	0.643	9.705	0.000
IES R (total)	0.034	−0.001	0.218	1.955	0.052
PSS-10 (steny)	0.376	−0.003	0.192	1.907	0.058
WAS (total)	−0.012	−0.107	0.056	−0.607	0.544
MODEL 2					
Intercept	4.052			1.596	0.112
AIS (insomnia vs. no insomnia)	1.471	0.139	0.329	4.853	0.000
GAD-7 (total)	0.600	0.416	0.632	9.584	0.000
IES R (total)	0.030	−0.013	0.206	1.731	0.085
PSS-10 (steny)	0.402	0.001	0.200	1.996	0.047
*WAS, The kindness of the surrounding world*	0.057	−0.026	0.148	1.377	0.170
*WAS, Sensibility and explainability of life events*	−0.049	−0.125	0.035	−1.112	0.268
*WAS, The value of human*	−0.090	−0.198	−0.012	−2.218	0.028
MODEL 3					
Intercept	4.323			1.672	0.096
AIS (insomnia vs. no insomnia)	1.536	0.148	0.340	5.031	0.000
GAD-7 (total)	0.616	0.428	0.646	9.716	0.000
IES R (total)	0.027	−0.024	0.197	1.544	0.124
PSS-10 (steny)	0.442	0.011	0.211	2.193	0.029
*WAS justice*	−0.021	−0.119	0.092	−0.246	0.806
*WAS, The kindness of people*	0.104	−0.041	0.148	1.119	0.265
*WAS Randomness*	0.151	−0.001	0.181	1.955	0.052
*WAS The World Kindness*	−0.128	−0.155	0.021	−1.496	0.136
*WAS, own value*	−0.155	−0.180	−0.013	−2.279	0.024
*WAS, happiness*	−0.081	−0.151	0.051	−0.984	0.326
*WAS Negative Event Control*	−0.010	−0.107	0.095	−0.113	0.910
*WAS, Controlling one’s own behavior*	−0.192	−0.194	−0.004	−2.055	0.041

b - regression coefficient, CI - confidence interval, ref - AIS reference level, Athens Insomnia Scale, GAD-7, generalized anxiety disorder; IES-R, Impact of Event Scale - Revised; PSS-10, The Perceived Stress Scale; WAS, The World Assumptions Scale.

Model 2, explains more than 66% of the variation in depression by the PHQ-9 [*F*(75, 199) = 60.561, *p* < 0.001]. Subjects exhibiting insomnia symptoms according to the AIS had higher levels of depression according to the PHQ-9 compared to those who had no such symptoms (*p* < 0.001). Moreover, it was observed that the higher the score obtained by the respondent in the subscale of the World Assumptions Scale - “human worthiness” the lower the level of depression according to the PHQ-9 (*p* = 0.028). The study also revealed that the higher the level of stress according to the PSS-10 scale, the higher the level of depression according to the PHQ-9 (*p* = 0.047; Table 4).

Model 3 explains more than 67% of the variation in the depressive variable according to the PHQ-9 [*F*(12, 194) = 36.089, *p* < 0.001]. Respondents with higher severity of generalized anxiety according to the GAD-7 scale were characterized by higher levels of depressiveness (*p* < 0.001). Likewise, subjects with insomnia symptoms according to the AIS had higher levels of depression as measured by the PHQ compared to subjects without such symptoms (*p* < 0.001). In addition, the higher the score obtained by the respondent in the subscale of the Assumptions Toward the World-” self-esteem scale” (*p* = 0.024) and “control of own behavior” (*p* = 0.041) the lower the level of depression measured by the PHQ-9 scale. The study also observed that the higher the level of stress according to the PSS-10 scale the higher the level of depression according to the PHQ-9 (*p* = 0.029; Table 4).

Model 4, explains more than 66% of the variation in depression by PHQ-9 [*F*(75, 199) = 60.561, *p* < 0.001]. Respondents with higher severity of generalized anxiety according to the GAD-7 scale were characterized by higher levels of depressiveness according to the PHQ-9 scale (*p* < 0.001). In addition, subjects with insomnia symptoms according to the AIS had higher levels of depression according to the PHQ-9 compared to those with no such symptoms (*p* < 0.001; Table 5).

**Table 5:** Influence of insomnia according to AIS, anxiety according to GAD-7, stress according to PSS-10, World Assumptions Scale on the prevalence of depressive symptoms among nurses according to PHQ-9 (Model 4,5,6).

Factors	*b*	−95% CI	+95% CI	*t*	*p*
MODEL 4					
Intercept	1.492			0.589	0.556
AIS (insomnia vs. no insomnia)	1.504	0.142	0.336	4.874	0.000
GAD-7 (total)	0.601	0.416	0.634	9.482	0.000
*IES R, Intrusion*	0.110	−0.017	0.293	1.759	0.080
*IES-R, Stimulation*	0.036	−0.146	0.219	0.394	0.694
*IES R, Avoiding*	−0.052	−0.184	0.078	−0.796	0.427
PSS-10 (steny)	0.377	−0.002	0.191	1.926	0.055
WAS (total)	−0.013	−0.109	0.054	−0.666	0.506
MODEL 5					
Intercept	4.233			1.656	0.099
GAD-7 (total)	0.588	0.405	0.622	9.320	0.000
*IES R, Intrusion*	0.086	−0.049	0.267	1.355	0.177
*IES-R Stimulation*	0.059	−0.124	0.243	0.643	0.521
*IES R, Avoiding*	−0.053	−0.185	0.078	−0.805	0.422
PSS-10 (steny)	0.389	−0.002	0.197	1.941	0.054
*WAS, The kindness of the surrounding world*	0.052	−0.034	0.145	1.219	0.224
*WAS, Sensibility and explainability of life events*	−0.060	−0.137	0.025	−1.356	0.177
*WAS, The value of human*	−0.080	−0.188	0.001	−1.948	0.053
MODEL 6					
Intercept	4.579			1.756	0.081
AIS (insomnia vs. no insomnia)	1.482	0.138	0.333	4.754	0.000
GAD-7 (total)	0.604	0.418	0.638	9.460	0.000
*IES R, Intrusion*	0.078	−0.061	0.257	1.210	0.228
*IES-R, Stimulation*	0.056	−0.128	0.241	0.606	0.545
*IES R, Avoiding*	−0.055	−0.188	0.076	−0.838	0.403
PSS-10 (steny)	0.429	0.008	0.207	2.133	0.034
*WAS, justice*	−0.017	−0.117	0.095	−0.205	0.838
*WAS, The kindness of people*	0.095	−0.046	0.143	1.017	0.310
*WAS, Randomness*	0.133	−0.013	0.172	1.687	0.093
*WAS, The World Kindness*	−0.136	−0.160	0.018	−1.580	0.116
*WAS, own value*	−0.162	−0.185	−0.017	−2.373	0.019
*WAS, happiness*	−0.075	−0.148	0.055	−0.903	0.368
*WAS, Negative Event Control*	−0.003	−0.105	0.101	−0.036	0.971
*WAS, Controlling one’s own behavior*	−0.178	−0.187	0.004	−1.897	0.059

b - regression coefficient, CI - confidence interval, ref - reference level, AIS- Athens Insomnia Scale, GAD-7-generalized anxiety disorder; IES-R, Impact of Event Scale - Revised; PSS-10 - The Perceived Stress Scale; WAS - The World Assumptions Scale.

On the basis of model 5 explaining more than 67% of the variation in depression variable according to the PHQ-9 [*F*(8, 198) = 47.334, *p* < 0.001], it was shown that the higher the level of generalized anxiety according to the GAD-7 scale, the higher the level of depression according to the PHQ-9 in the study group (*p* < 0.001; Table 5).

Model 6 explains more than 67% of the variation in depression by the PHQ-9 [*F*(14, 192) = 30.985, *p* < 0.001]. This analysis showed that the higher the GAD-7 scale score, the higher the level of depression according to PHQ-9 (*p* < 0.001). Respondents showing insomnia symptoms according to AIS have higher levels of depression according to PHQ-9 (*p* < 0.001). In addition, the higher the score obtained by the respondent on the subscale of the Assumptions Toward the World-” self-esteem scale” (*p* = 0.019) the lower the level of depression measured by the PHQ-9 scale. The study also observed that the higher the level of stress according to the PSS-10 scale the higher the level of depression according to the PHQ-9 (*p* = 0.034; Table 5).

Based on model 7 explaining more than 65% of the variability of the depressive variable according to PHQ-9 [*F*(8, 198) = 50.231, *p* < 0.001]. Respondents with mild anxiety symptoms according to GAD-7 show significantly lower levels of depression as measured by PHQ scores compared to those who do not experience anxiety symptoms (*p* < 0.001). Respondents with moderate and severe anxiety symptoms according to GAD-7 are characterized by higher levels of depression according to PHQ-9 in comparison to persons who do not experience anxiety symptoms, and the observed relationship is stronger in the case of severe anxiety symptoms (*p* = 0.022 and *p* < 0.001). Respondents with insomnia symptoms according to the AIS score higher on the PHQ-9 scale compared to those without such symptoms (*p* < 0.001). In model 7 it was also noted that the higher the level of perceived stress according to PSS-10 (*p* = 0.045) or higher scores obtained in the IES-R scale (*p* = 0.005) the higher the level of depression according to PHQ-9 (Table 6).

**Table 6:** Influence of insomnia according to AIS, anxiety according to GAD-7, stress according to PSS-10, World Assumptions Scale on the prevalence of depressive symptoms among nurses according to PHQ-9 (Model 7,8,9).

Factors	*b*	−95% CI	+95% CI	*t*	*p*
MODEL 7					
Intercept	8.970			3.665	0.000
AIS (insomnia vs. no insomnia)	1.709	0.176	0.367	5.617	0.000
*GAD-7 no anxiety (ref.)*					
*GAD-7, mild anxiety*	−2.809	−0.497	−0.268	−6.593	0.000
*GAD-7, moderate anxiety*	1.175	0.020	0.254	2.312	0.022
*GAD-7, severe anxiety*	5.988	0.446	0.734	8.087	0.000
IES R (total)	0.049	0.048	0.262	2.847	0.005
*PSS-10, low stress level (ref.)*					
*PSS-10, average stress level*	−0.422	−0.116	0.048	−0.823	0.412
*PSS-10, high stress level*	0.949	0.002	0.179	2.017	0.045
WAS (total)	−0.011	−0.107	0.058	−0.587	0.558
MODEL 8					
Intercept					
AIS (insomnia vs. no insomnia)	1.599	0.155	0.353	5.071	0.000
*GAD-7, no anxiety (ref.)*					
*GAD-7 mild anxiety*	−2.705	−0.486	−0.251	−6.199	0.000
*GAD-7, moderate anxiety*	1.145	0.016	0.251	2.242	0.026
*GAD-7, severe anxiety*	5.804	0.425	0.720	7.646	0.000
*IES R, Intrusion*	0.093	−0.044	0.277	1.428	0.155
*IES-R, Stimulation*	0.042	−0.147	0.231	0.439	0.661
*IES R, Avoiding*	−0.001	−0.137	0.135	−0.012	0.990
*PSS-10, low stress level (ref.)*					
*PSS-10, average stress level*	−0.355	−0.111	0.054	−0.686	0.493
*PSS-10, high stress level*	0.994	0.005	0.185	2.082	0.039
*WAS, The kindness of the surrounding world*	0.044	−0.044	0.138	1.021	0.309
*WAS, Sensibility and explainability of life events*	−0.051	−0.131	0.036	−1.117	0.266
*WAS, Value of human*	−0.072	−0.180	0.011	−1.743	0.083
MODEL 9					
Intercept	11.537			4.492	0.000
AIS insomnia vs. no insomnia	1.660	0.163	0.364	5.174	0.000
*GAD-7, no anxiety (ref.)*					
*GAD-7 mild anxiety*	−2.734	−0.494	−0.251	−6.048	0.000
*GAD-7 moderate anxiety*	1.224	0.024	0.261	2.372	0.019
*GAD-7, severe anxiety*	5.840	0.425	0.726	7.549	0.000
*IES R, Intrusion*	0.090	−0.050	0.276	1.363	0.175
*IES-R, Stimulation*	0.041	−0.151	0.234	0.423	0.673
*IES R, Avoiding*	−0.004	−0.142	0.133	−0.064	0.949
*PSS-10, low stress level (ref.)*					
*PSS-10, average stress level*	−0.242	−0.104	0.064	−0.459	0.647
*PSS-10, high stress level*	1.042	0.009	0.190	2.170	0.031
*WAS, justice*	−0.065	−0.149	0.068	−0.742	0.459
*WAS, The kindness of people*	0.088	−0.052	0.142	0.914	0.362
*WAS, Randomness*	0.091	−0.041	0.149	1.121	0.264
*WAS The kindness of the world*	−0.065	−0.126	0.058	−0.722	0.471
*WAS, own value*	−0.138	−0.172	0.001	−1.957	0.052
*WAS, happiness*	−0.060	−0.143	0.069	−0.692	0.490
*WAS, Negative Event Control*	−0.019	−0.118	0.096	−0.201	0.841
*WAS, Controlling one’s own behavior*	−0.108	−0.153	0.042	−1.127	0.261

b - regression coefficient, CI - confidence interval, ref - reference level, AIS- Athens Insomnia Scale, GAD-7-generalized anxiety disorder; IES-R, Impact of Event Scale - Revised; PSS-10 - The Perceived Stress Scale; WAS - The World Assumptions Scale.

In the case of model 8 explaining 66% of the variability of the depressive variable according to the PHQ-9 [*F*(12, 194) = 34.105, *p* < 0.001], it was shown that respondents with high levels of perceived stress according to the PSS-10 show significantly higher levels of depression according to the PHQ-9 compared to those who do not feel stress (*p* = 0.039). Nurses with mild anxiety symptoms according to GAD-7 were observed to show significantly lower levels of depression as measured by PHQ-9 scores compared to those who do not experience anxiety symptoms (*p* < 0. 001), while respondents with moderate and severe anxiety symptoms according to GAD-7 showed significantly higher levels of depression measured by PHQ scores compared to those who do not experience anxiety symptoms, and the observed relationship was stronger for severe anxiety symptoms (*p* = 0.026 and *p* < 0.001). Furthermore, respondents with insomnia symptoms according to the AIS have higher levels of depression as measured by PHQ scores compared to those without such symptoms (*p* < 0.001; Table 6).

The last model explaining 65% of the variation in the depressive variable [*F*(17, 189) = 23.861, *p* < 0.001], proves that nurses with high levels of perceived stress according to PSS-10 have significantly higher levels of depression as measured by PHQ scores compared to those who do not experience stress (*p* = 0.031). In addition, respondents with mild anxiety symptoms according to the GAD-7 show significantly lower levels of depression according to the PHQ-9 compared to individuals who do not experience anxiety symptoms (*p* < 0.001). Respondents with moderate and severe anxiety symptoms according to GAD-7 showed significantly higher level of depression in comparison to persons who do not experience anxiety symptoms, and the observed relationship was stronger in the case of severe anxiety symptoms (*p* = 0.019; *p* < 0.001). Respondents with insomnia symptoms according to the AIS score higher on the PHQ-9 scale compared to those without such symptoms (*p* < 0.001; Table 6).

## 4. Discussion

The mental health of nurses during the COVID-19 pandemic is important, as it can affect their productivity and reduce the quality of care provided. Unfortunately, there have been several reports of suicides among health workers due to psychological pressure and possible fear of death (30). In view of the critical situation of medical staff directly dealing with various aspects of COVID-19 (diagnosing, as well as care and therapy), many studies confirm that this group carries a heavy burden of mental distress and physical overload. In addition, previous studies conducted during the SARS outbreak in 2003 confirm negative psychological reactions in relation to unknown disease entities (31).

### 4.1. Analysis of perceived stress, assumptions about the world and the presence of anxiety, depression, and insomnia among nursing staff during the SARS-CoV-2 coronavirus pandemic

In our study, PSS-10 scale was used to assess stress among the nurses, where the mean scores presented in stens were 7.14 (SD = 1.56). It was observed that as many as 72.95% of the respondents showed high levels of stress. When the results of Generalized Anxiety Questionnaire (GAD-7) were analyzed, the mean score obtained was 7.12 (SD = 5.41). In 65.7% of the respondents, the total score indicated the presence of anxiety symptoms of varying degrees of severity. In case of the PHQ-9 scale, the mean score among the respondents was 7.77 (SD-6.20) and 37.2% of the respondents had no symptoms of depression. On the other hand, the analysis of AIS results showed that the mean score on the scale was 6.71 (SD-4.39), and 59.42% of the respondents had no insomnia. In our study, the psychological impact of the COVID-19 pandemic, assessed by the IES-R scale, corresponded with an average score of 34.25 (SD = 19.65), and the World Assumptions Scale showed that the highest mean score was for happiness (*M* = 17.52), control of one’s own behavior (*M* = 16.77), and kindness of people (*M* = 15.88), while the most dominant group assessed in the WAS scale was human worthiness (*M* = 50.48).

Gawrych’s (32) study showed that the pandemic exacerbates symptoms of depression, anxiety, psychological distress and affects sleep quality. It is also significant that reduced well-being and higher anxiety and depression scores were observed among the subjects compared to the pre-COVID-19 period. Also, a study by Shaukat et al. (33) confirms that frontline workers are exposed to physical and psychological consequences as a direct result of providing care to patients with COVID-19, which further exacerbates stress. Results from Du et al. (34) suggest that frontline health workers in Wuhan were under moderate to severe stress at the peak of the outbreak, with many reporting elevated anxiety and depression. A study by Wang et al. (35) found that during the COVID-19 outbreak, 38% of pediatric health care workers suffered from sleep disturbances, while 7% had anxiety and 25% had depressive symptoms. Furthermore, it was observed that sleep disturbances experienced by medical professionals were independently related to being an only child, depression, and contact with those with COVID-19 infection.

Vindegaard et al. (36) also informed that medical staff experienced increased symptoms of depression and psychological distress due to the COVID 19 pandemic. Among medical staff, only 36.3% received written psychoeducational materials (brochures, leaflets, books), psychological support through the media (online psychological support), or participated in group psychological counseling (17.5%). Because of the burden placed on health care workers, there is an increasing need to protect them by providing personal protective equipment, training, and counteracting fatigue (37). In a cross-sectional study conducted in China on a sample of 7,236 subjects, including nurses and other health care workers, there were high scores indicating the prevalence of generalized anxiety syndrome, depression and poor sleep quality (38).

A meta-analysis by Pappa et al. (39), confirmed high levels of psychological stress, anxiety, and depression among healthcare personnel. Whose meta-analysis showed that the overall incidence of anxiety was 24.06% (95% CI 16.84–32.09, I2 = 99%) and depression–22.8% (95% CI 15.1–31 51, I 2 = 99.62).

A growing body of scientific literature provides new evidence of the significant impact of the COVID-19 pandemic on the psychological functioning of medical professionals. A study by Cheung et al. (40) conducted in Hong Kong indicates that medical staff are susceptible to occupational burnout, anxiety and mental exhaustion. On the other hand, severe symptoms of anxiety and depression were observed in German physicians (10). What is particularly disturbing, suicide attempts and suicides have also been reported among health care workers facing cumulative psychological stress and increased death concern. This is a significant phenomenon, since physicians are at higher risk of suicide than the general population (30).

### 4.2. Analysis of the relationship between mental health variables (anxiety, stress, insomnia) and levels of depression among nurses during the SARS-CoV-2 coronavirus pandemic

There are many factors involved in the development of depression: individual, demographic, work-related, and environmental. The occurrence of depression can also be ascribed to the lack of resilience, ineffective coping strategies, and sleep problems. Our study showed that the main factors contributing to depression in the study group were high levels of anxiety, stress and insomnia.

A study by Zhu et al. (41). showed that a history of depression or anxiety was a factor predisposing nurses to anxiety and depressive symptoms. Lai et al. (42), on the other hand, found that Wuhan health workers had more severe symptoms of depression, anxiety, insomnia and stress compared to Hubei workers. These results indicate that the risk of depression and anxiety symptoms was greater at the epicenter of the epidemic in China than outside this area. Logistic regression analysis by Wang et al. (35) demonstrated that being an only child, having contact with people infected with COVID-19, and sufferring from depression were factors associated with the occurrence of sleep disorders.

Systematic review and meta-analysis by Al Maqbali (43) reporting pooled prevalence estimates for stress, anxiety, depression and sleep disturbance among nurses during the COVID-19 outbreak. The findings show that over one third of nurses have experienced stress, anxiety, depression and sleep disturbance during the COVID-19 outbreak, which is higher than the previous Middle East respiratory syndrome (MERS) and severe acute respiratory syndrome (SARS) epidemics. Similar systematic review and meta-analysis by Ślusarska (44) provide a long-term and comprehensive synthesis of existing evidence confirming the incidence of depressive disorders in more than one-fifth of those studied, and anxiety symptoms among just under one-third of nurses, during the COVID-19 pandemic.

A cross-sectional replication study conducted 1 year after the COVID-19 outbreak to assess the impact of the health workers (*n* = 1,033) at a teaching hospital in Verona (Italy) showed, that the proportion of healthcare workers above the cut-off point increased from 2020 to 2021 in all performance domains (anxiety, 50.1% vs. 55.7, *p* < 0.05; depression, 26.6% vs. 40.6%, *p* < 0.001). In contrast, multivariate analysis showed that 1 year after the COVID-19 outbreak, nurses were more likely to experience anxiety and depression than other healthcare professionals (45).

Pang et al. (46) noted that positive coping strategies and good quality of sleep were accompanied by lower levels of anxiety and depressive symptoms. Quite the reverse, using the mechanisms of passive coping was linked to a worse mental state.

Analogous findings were presented by Mei et al. (47), who found sleep disturbances to be a significant predictor of anxiety and depressive symptoms among nurses fighting the pandemic, which was mainly due to excessive work and clinical workload. Quality of sleep may be of great importance in the context of anxiety and depression. It has been proven that the right amount of good quality sleep can be a protective factor against mental illnesses (48), and that people with sleep disorders are more prone to anxiety and depression (49). Sleep disorders are a very strong biological indicator of depression. Although insomnia usually begins the development of depression in a more distant perspective, insomnia can also immediately precede the onset of depressed mood, or occur with it at different times. Insomnia, on the other hand, may be the body’s response to stress or traumatic events (50).

In summary, stress, anxiety, depression and sleep disorders are significant problems for nurses worldwide during an infectious disease outbreak. Organizations should provide support services counseling or online workshops and training materials to enable them to overcome any psychological problems. In addition, they should improve the working conditions of nurses by increasing the manpower and resource allocation. Nurse managers play a key role through effective communication, rotating nurses, implementing flexible schedules and encouraging nurses to the use of psychosocial and psychological support services (51–55).

## 5. Limitations

While the literature of the subject is still sparse, several studies analyzing contributors to depression among healthcare workers during the COVID-19 pandemic have already been published. As far as we know, ours is one of the first studies conducted among Polish nurses. Sorry to say, our study is not free from limitations. One of them is a rather extensive questionnaire, which is not an element that encourages overworked nurses to participate in the study. Another problem is that our study was cross-sectional, therefore, we cannot say whether depressive symptoms occurred only during the COVID-19 pandemic or were already present before. Furthermore, we are unable to determine what the severity of depressive symptoms will be if the COVID-19 pandemic continues even longer. Therefore, it is important to conduct further research after the COVID-19 pandemic has ended.

## 6. Recommendations for further research

The COVID-19 pandemic affected the psychological health of medical staff, particularly through increased stress and anxiety symptoms. It is important to conduct further research after the COVID-19 pandemic has ended. Its findings can be useful in planning prophylactic measures and adapting intervention strategies aimed at applying nurse-motivation-supporting techniques targeted at pro-health behaviors. Health practices are an area requiring special measures. It is worth considering the possibility of providing institutional support and psychological aid to nurses.

## 7. Conclusion

Nursing staff were at high risk of experiencing traumatic stress, anxiety, insomnia, and depressive symptoms of varying severity during the COVID-19 pandemic.

As stress, anxiety, and insomnia increased in the study nurses, the risk of depressive symptoms increased. In contrast, the higher the level of self-esteem and self-behavioral control, the lower the level of depressive symptoms.

## Data availability statement

The raw data supporting the conclusions of this article will be made available by the authors, without undue reservation.

## Ethics statement

The studies involving humans were approved by Bioethics Committee of the Pomeranian Medical University in Szczecin. The studies were conducted in accordance with the local legislation and institutional requirements. The participants provided their written informed consent to participate in this study.

## Author contributions

KR: Conceptualization, Formal Analysis, Methodology, Supervision, Visualization, Writing – original draft, Writing – review & editing. MP: Validation, Writing – review & editing. MS: Resources, Writing – review & editing. PU: Investigation, Writing – review & editing. EG: Funding acquisition, Project administration, Writing – review & editing. AC: Conceptualization, Formal Analysis, Investigation, Methodology, Project administration, Visualization, Writing – original draft, Writing – review & editing.

## Funding

The authors declare that no financial support was received for the research, authorship, and/or publication of this article.

## Conflict of interest

The authors declare that the research was conducted in the absence of any commercial or financial relationships that could be construed as a potential conflict of interest.

## Publisher’s note

All claims expressed in this article are solely those of the authors and do not necessarily represent those of their affiliated organizations, or those of the publisher, the editors and the reviewers. Any product that may be evaluated in this article, or claim that may be made by its manufacturer, is not guaranteed or endorsed by the publisher.

## Footnotes

^1^https://docs.google.com/; Accessed on 21 November 2021.

^2^ www.phqscreeners.com
